# A novel protocol for rapid deployment of heart rate data storage tags in Atlantic bluefin tuna *Thunnus thynnus* reveals cardiac responses to temperature and feeding

**DOI:** 10.1111/jfb.15507

**Published:** 2023-08-03

**Authors:** T. Rouyer, S. Bonhommeau, S. Bernard, V. Kerzerho, O. Derridj, Á. Bjarnason, H. Allal, J. F. Steffensen, S. Deguara, B. Wendling, G. Bal, D. Thambithurai, D. J. Mckenzie

**Affiliations:** ^1^ MARBEC University of Montpellier, CNRS, IFREMER, IRD Sète France; ^2^ IFREMER DOI, Rue Jean Bertho Le Port France; ^3^ LIRMM, University of Montpellier, CNRS Montpellier France; ^4^ Star‐Oddi Ltd. Garðabær Iceland; ^5^ University Hospital of Montpellier Montpellier France; ^6^ Marine Biological Section University of Copenhagen Denmark; ^7^ AquaBioTech Group, ‘Central Complex’ Mosta Malta; ^8^ SATHOAN Sète France; ^9^ UMS PatriNat (OFB‐CNRS‐MNHN) Brunoy France

**Keywords:** Atlantic bluefin tuna, heart rate, longliner, physiology, protocol, tagging

## Abstract

The Atlantic bluefin tuna (ABFT) is a highly prized species of large pelagic fish. Studies of their environmental physiology may improve understanding and management of their populations, but this is difficult for mature adults because of their large size. Biologging of heart rate holds promise in investigating physiological responses to environmental conditions in free‐swimming fishes but it is very challenging to anesthetize large ABFT for invasive surgery to place a tag in the body cavity near to the heart. We describe a novel method for rapid deployment of a commercially available heart‐rate tag on ABFT, using an atraumatic trocar to implant it in the musculature associated with the cleithrum. We performed three sequential experiments to show that the tagging method (1) is consistently repeatable and reliable, (2) can be used successfully on commercial fishing boats and does not seem to affect fish survival, and (3) is effective for long‐term deployments. In experiment 3, a tag logged heart rate over 80 days on a 60‐kg ABFT held in a farm cage. The logged data showed that heart rate was sensitive to prevailing seasonal temperature and feeding events. At low temperatures, there were clear responses to feeding but these all disappeared above a threshold temperature of 25.5°C. Overall, the results show that our method is simple, rapid, and repeatable, and can be used for long‐term experiments to investigate physiological responses by large ABFT to environmental conditions.

## 1 INTRODUCTION

The Atlantic bluefin tuna (*Thunnus thynnus*, hereafter ABFT) is a large and highly migratory pelagic species that supports a valuable international fishery (Fromentin & Powers, 2005). For ABFT, understanding the interplay between environmental conditions and migrations has important implications in terms of the species' exploitation and conservation in a context of global change (Rooker et al., 2007; Fromentin, Reygondeau, et al., 2014). Studies of tuna environmental physiology may provide insights into drivers of migratory movements (Block et al., 2001, 2011; Brill, 1994; Clark et al., 2013; Fry, 1971; Horodysky et al., 2016; Whitlock et al., 2015). Studying the in vivo physiology of mature adult ABFT is, however, logistically very challenging and costly, especially if it involves invasive techniques, due to their size, highly active lifestyle with obligate ram ventilation of the gills, and sensitivity to handling. Such challenges largely explain why physiological studies on bluefin tuna species are scarce and generally involve experiments on small individuals (Blank et al., 2007a,b; Block et al., 2001, 2005; Clark et al., 2013).

For large animals, one option is to work with partners in the private sector, fishermen and fish farmers, and use techniques such as biologging to gain physiological information on animals under field conditions (Rouyer et al., 2019). Electronic data storage tags can log heart rate from an electrocardiogram (ECG) and have been used to record cardiac responses to environmental conditions by free‐swimming fishes including a bluefin tuna species (Brijs et al., 2019; Clark et al., 2008; Gamperl et al., 2021; Mignucci et al., 2021). These previous studies all worked on relatively small fishes and placed the tags in the peritoneal cavity (Brijs et al., 2019; Clark et al., 2008; Gamperl et al., 2021; Mignucci et al., 2021). Such a surgical intervention on large ABFT would be extremely difficult, especially if it is from the deck of a commercial vessel, with a risk of harming the fish and obtaining little or no meaningful data.

Here, we propose a novel method for implantation of a heart‐rate tag (HR‐tag) that is rapid, easy and relatively noninvasive, which can simplify tagging of ABFT under field conditions, such as during commercial fishing or farm husbandry operations. The HR‐tag is inserted by trocar into the musculature associated with the cleithrum, where this membrane bone runs below the operculum and in close proximity to the heart. We describe three sequential experiments on live ABFT to validate the methodology. The first experiment tested the feasibility of the HR‐tag deployment and the quality of the signals obtained on freshly captured tuna on the deck of a commercial longliner. The second experiment assessed the fate of fish implanted on the deck of a longliner and then released with survival tags. The third experiment tested whether or not the technique was suitable for long‐term deployment by implanting ABFT tuna on a fish‐farming barge and then releasing them into a rearing cage for several months. The HR data collected in the third experiment was analyzed to explore whether temperature and the amount of food provided were associated with variations in cardiac activity. Our findings are discussed in terms of the usefulness of the technique to study ABFT physiology.

## 2 MATERIALS AND METHODS

### 2.1 Ethics statement

Experimental procedures were approved by the Ethics Committee for Animal Experimentation No. 036 of the French Ministère de l'Enseignement Superieur, de la Recherche et de l'Innovation, with reference number APAFIS #32610‐2021062115252354 v7.

### 2.2 Tagging protocol

**FIGURE 1:**
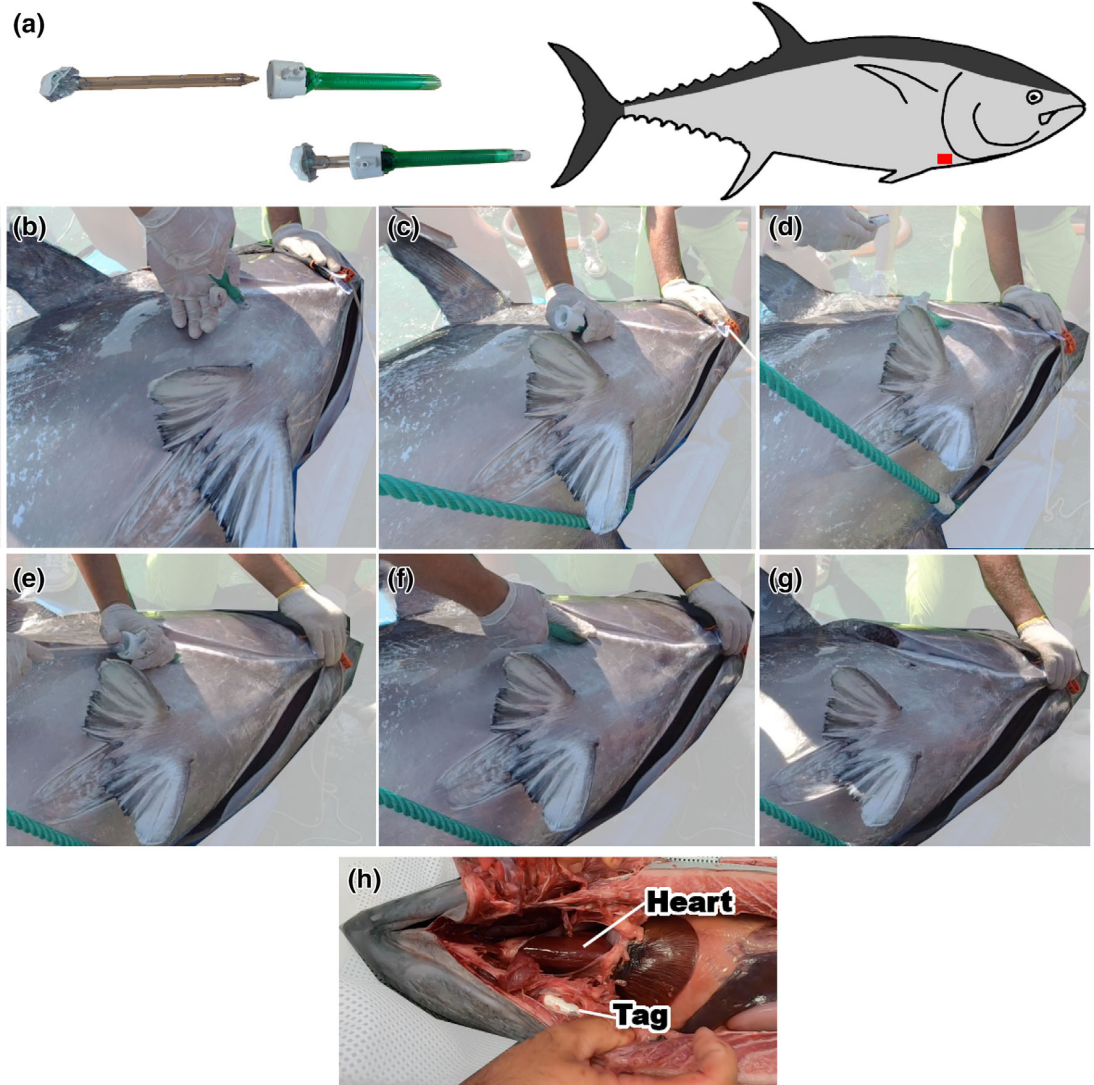
Material used for implanting HR‐tags (a), description of a HR‐tag deployment in a live ABFT (b–g), and dissection of a dead Atlantic bluefin tuna (ABFT) implanted with a HR‐tag (h). (a) The atraumatic trocar that was used for the implantation, with the removable obturator, which is then used to push the HR‐tag into the fish. The entry point for the trocar is below the point of maximum curvature of the operculum, into muscles associated with the cleithrum, a membranous bone (b). Once the trocar is inserted and advanced forward in the fatty flesh, the obturator is removed (c) and the tag placed into the outer cannula (d) and advanced with the obturator (e) into the tissue (f). The trocar is then removed (g) and the fish is released back into the water. In (h), a dead tuna was implanted with a tag following this protocol, then dissected ventrally to show the tag location in close proximity to the heart.

Tagging was performed on conscious fish on the deck of a vessel, with fish placed on a large plastic‐covered mattress, their eyes covered with a cloth and gills force‐ventilated with a flow of seawater. ABFT show tonic immobility when air‐exposed and placed on their side, facilitating tagging procedures. The site of tagging was the fleshy area below the operculum, into muscles associated with the cleithrum that, in ABFT, are in close proximity to the heart, is rich in fat and thick enough to hold an implanted tag (Figure 1a). Tag placement was by trocar (Patel et al., 2019) with a cannula that had an internal diameter similar to the tag diameter. We used a disposable 15 × 150 mm atraumatic trocar that allows wound healing without sutures (Kii Fios First Entry Trocar, Applied Medical). First, a small incision was made in the skin (10 mm diameter, 5 mm deep) a couple of centimeters under the point of maximum curvature of the operculum (Figure 1a,b). The trocar was sterilized in a bath of chlorhexidine and then inserted into the incision and advanced parallel to the lower edge of the operculum, where it followed a groove in the cleithrum, within the thickness of the flesh (Figure 1b). The depth of insertion had to be carefully considered depending on the size of the fish to ensure that the trocar remained within the muscle and did not risk damaging or traversing the membranous cleithrum, which may also lead to damage of underlying blood vessels or the heart in smaller individuals. As a rule of thumb, the depth of insertion was at least 2 cm to allow the flesh to close back together completely as the trocar was withdrawn, which it was hoped would promote rapid healing. The obturator of the trocar was then removed and the cylindrical HR‐tag (Star‐Oddi® Centi‐HRT‐ACT) inserted into the open cannula (Figure 1c,d), then the obturator was used to advance the tag gently into the tissue. The whole trocar was then removed, the flesh closed completely behind the trocar, and no suture was used to close the point of entry as this would increase handling time with potential negative effects on fish recovery.

### 2.3 Experiment 1: Efficacy of the method

The tagging protocol was tested on three live ABFT caught during a French commercial longliner set in the Gulf of Lions (Rouyer et al., 2021). Longliners operate on short sets and numerous ABFT are still alive when hauled onboard, providing the opportunity to perform rapid and brief tag deployments. The ABFT fishery is strictly regulated, and fish are killed immediately after they have been hauled onboard.

The fish handling protocol has been described in detail previously (Rouyer et al., 2019, 2020). Three Centi‐HR‐ACT tags were programmed using Mercury software 5.99 (Star‐Oddi) to continuously record HR every other minute. Three fish were implanted, the first (HR‐tag1, 120 cm SFL) had just been killed by the Ike Jime technique (Pérez‐Lloréns, 2019) but was not bled to maximize the chances that the heart would still be beating. The other two (HR‐tag2 126 cm and HR‐tag3 152 cm) were implanted alive. The tags were left in the fish for about 3 min then animals were killed and/or bled by the fishermen, and tags retrieved. Data were immediately downloaded and the quality of ECG signals visually inspected using Star‐Oddi Pattern Finder software.

### 2.4 Experiment 2: Survival of tagged fish

The potential effect of HR‐tag deployment on mortality was tested on three ABFT (138, 149, and 116 cm) fitted with survival tags (Spat, Wildlife Computers) during the same fishing operation as for Experiment 1. The Spat tags were deployed as described previously (Rouyer et al., 2019, 2020). The third fish was also implanted by trocar with a nonfunctional Centi‐HR‐tag to compare the survival of implanted and nonimplanted fish. Death of the animal causes tag release and they were set to release after 50 days, which would indicate survival until at least that time. Information about the area of release was received via the Argos system. The time out of the water was below 2 min for all fish, which were then immediately released back into the wild.

### 2.5 Experiment 3: Long‐term deployment of HR‐tag

To test the suitability of the tagging protocol for longer deployments studies, we implanted three fish in a fattening cage in Malta with custom‐made Star‐Oddi DST centi‐HRT ACT G2 (70 mm long, 15 mm diameter, 26.5 g weight in air) and placed a spaghetti tag on the dorsal fin for visual identification. The fish spent less than 3 min out of the water and were then released back into the cage.

The tags were custom‐made to allow more memory and a larger battery. They were programmed so that each 10 min they recorded the ECG for 7.5 s at 200 Hz, tri‐axial acceleration for 1 min at base frequency 25 Hz, and temperature. On‐board algorithms within the tags calculated HR and an associated quality index (QI), and various statistical variables for external acceleration (EA) every 10 min. EA is the recorded three‐axis acceleration above standard gravity defined with static calibration, normalized, and calculated as the vectorial sum in milli‐g (mg). The approach is similar to both the calculation of the vectoral sum of body acceleration (VeDBA) (Qasem et al., 2012; Shepard et al., 2008) and the minimum specific acceleration (MSA) (Simon et al., 2012). We report the average activity values (AvgEA). Raw data for ECG and acceleration were stored once every 30 min.

The quality of the ECG traces used to calculate HR was verified using HRT‐Analyzer software (v. 1.1.0, Star‐Oddi) as the QI, which ranged from QI = 0 (Excellent) to QI = 3 (Poor), with visual inspection of ECG traces to confirm the HR values returned by the algorithm. HR measurements with QI = 3 and 2 were eliminated as well as HRs above 120 bpm or below 8 bpm, this latter being the lower theoretical limit of HR measurable over a 7 s interval (see Supporting Information Data S1).

Data for each variable were then summarized over each day as the median heart rate (HRm), peripheral body temperature (TPm), and acceleration (ACm), and the variability in temperature over each day was measured through its interquartile range (TPiq). The daily total food fed to the cage was recovered from the fish farm to further interpret daily variations in HR.

The variations in HRm in relation to total food given to the cage (TF), TPm, TPiq, and ACm were investigated using a model selection approach with a set of nested linear models. The set of models that we considered to assess whether the variables had a statistically significant impact on HRm can be summarized as follows:

(1) $$\left\{\begin{array}{c} \mathrm{HR}m_{t}∼\text{Poisson}\left(\mu_{t}\right)\\ \mu_{t}∼\alpha +\beta_{i}\times X_{i,t} \end{array}\right.$$

where *X*_*i*_ denotes the *i*th variable included and $\beta_{i}$ is the parameter linking it linearly to HRm.

The use of a Poisson over a gamma error model was decided after some preliminary analyses and for the sake of parsimony as it allows for one parameter less (details not shown). Temperature has profound physiological effects on fishes that are often not linear (Fry, 1971; Schulte et al., 2011), so we decided to test for temperature thresholds associated with a change in the intensity and/or polarity of the link between HRm and the variable considered. To do this, for each given temperature variable TX we refined the second line of equation set (1) as:

$$\mu_{t}∼\alpha +\beta_{\mathrm{TX}}\times TX_{t}$$

$$\text{with}\;\beta_{\mathrm{TX}}=\left\{\beta_{\mathrm{TX}}^{+}\;\text{if}\;{\mathrm{TX}}_{t}>{\mathrm{TX}}_{\text{thresh}}\right\}\left\{\beta_{\mathrm{TX}}^{-}\;\text{if}\;{\mathrm{TX}}_{t}<{\mathrm{TX}}_{\text{thresh}}\right\}$$

Here we considered all the possible combinations of the variables TF, TPm, TPiq, and ACm but limited the complexity to only one threshold. We developed this nested modeling in a Bayesian framework because it provided (i) a formal and flexible framework to estimate threshold effects and (ii) estimates of parameter uncertainties in a fully probabilistic framework. The nested models were compared using two criteria. The first was the Deviance Information Criterion (DIC; Spiegelhalter et al., 2002), whose rationale is comparable to that of Akaike information criterion in that it allows the comparison of goodness of fit while penalizing complexity. The smaller the DIC the better and a five‐point reduction is usually considered as indicative of a significant improvement. Second, we reinforced the selection of significant variables by looking at the percentage of variance explained by each model through their *R* ^2^.

Models were implemented with JAGS software (Plummer, 2003) through the library Rjags in R (R Development Core Team, 2022). Specifically, we ran three Markov Chain Monte Carlo (MCMC) in parallel for each model fit. We kept 10,000 draws for each one after an initial burning period of 10,000 draws. All the parameters used were given noninformative priors. MCMC mixing and convergence were established by visual inspection of the chains' histories and by means of Brooks–Gelman–Rubin statistics (Brooks & Gelman, 1998). The code and data are available on request to the authors.

## 3 RESULTS

### 3.1 Experiments 1 and 2

**FIGURE 2:**
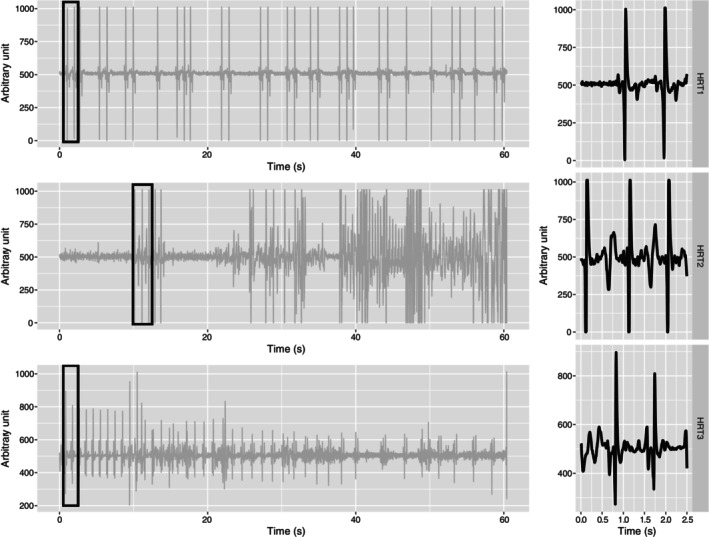
One minute of HR data acquired from Experiment 1. The three rows represent HR‐tag1 (top), HR‐tag2 (middle), and HR‐tag3 (bottom). The box identifies the chunk of data presented in detail in the right‐hand column, which depicts individual electrocardiogram (ECG) waveforms identified within the signal.

**FIGURE 3:**
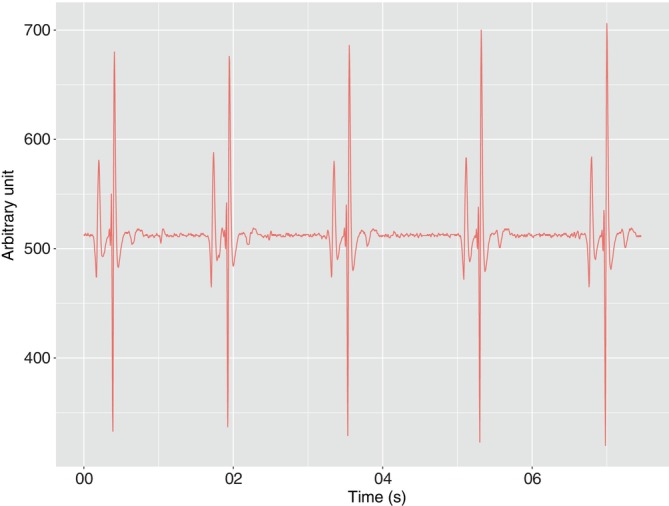
A sample electrocardiogram (ECGs) of 7 s duration, corresponding to 43 bpm, recorded on a 60‐kg fish tagged and released into a rearing cage.

The tag implantation only caused limited hemorrhaging and the briefly recorded data displayed a high signal to noise ratio. HR‐tag1 displayed a signal identifiable as an ECG (Figure 2). HR‐tag2 and HR‐tag3 recorded some signals that were identifiable as ECGs (Figure 2) although, overall, the data were not uniform and less clear than HR‐tag1. Two of the three survival tags reported after 50 days, one off to the south‐east of Majorca from the fish implanted with the HR‐tag, the other near the western coast of Sardinia (Figure 3).

### 3.2 Experiment 3

This experiment confirmed that implanted ABFT do not show subsequent mortality as two were slaughtered during farming operations and the last one is currently still alive in the cage. The tag was retrieved successfully from ABFT5, HR‐tag5 (mass 60 kg), at slaughter 80 days after deployment. Of 11,368 measurements collected, 9680 passed quality control, representing 85%.

These data showed a very clear signal with repeated waveforms and elements that are discernable as PQRST components (Figure 3). The waveform was generally well‐preserved across measurement samples and variations in the ECG are clearly visible among the 7‐s recordings. The data were generally much less noisy than those obtained in Experiment 1, with less noise and clearer ECGs.

**FIGURE 4:**
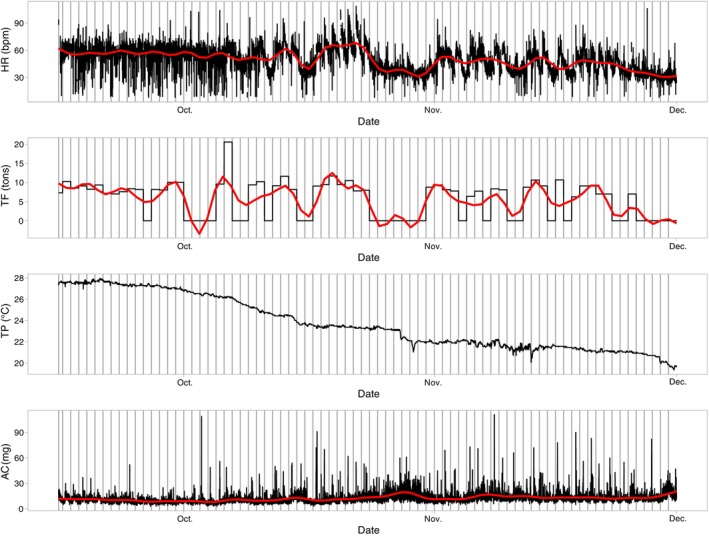
Time series of the raw data for heart rate tag (HR) (in bpm, top panel), total feed provided to the cage (TF in tons, second panel), peripheral body temperature logged on the tag (TP in °C, third panel), and average activity values (AC in mg, bottom panel). The different days are separated by vertical black lines. A smoother was added in red to the time series of HR, TF, and AC to help visualize trends.

**FIGURE 5:**
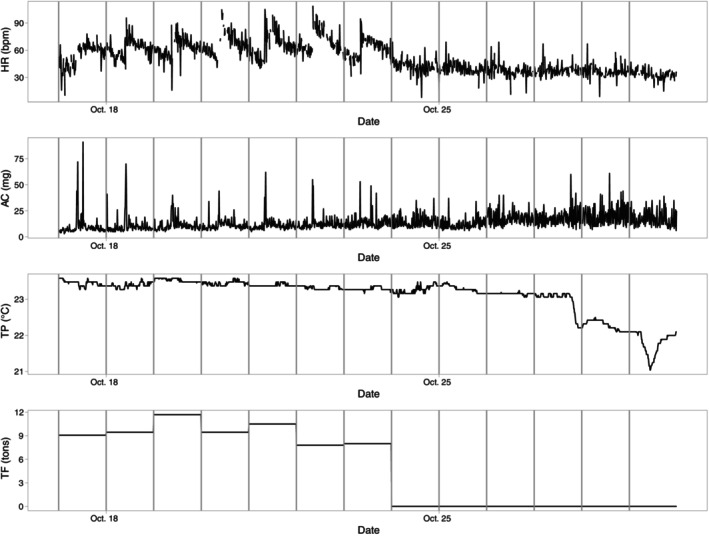
Time series of raw heart rate data (HR in bpm, top panel), activity (AC in mg, second panel), temperature (TP in °C, third panel), and total food (TF in tons, bottom panel) over 13 consecutive days. Colors indicate different days separated by vertical black lines.

All variables monitored during the experiment showed variations but also trends. HR ranged between 8 and 109 beats per minute (bpm) but 90% of the data were between 29 and 70 bpm, with a median of 50 bpm. Supporting Information Figure S1 shows a frequency distribution of HR measures, indicating a bimodal distribution with two peaks at 35–40 and 55–60 bpm. There was a general downward trend in HRm over time (Figure 4), but clear daily variations could be seen over certain time periods (Figure 5). Meanwhile, the TF also displayed strong variations, ranging from 0 kg up to 20 tons. Over the deployment period, the TPm decreased from about 28 to 20°C, with variation that was less marked than for the other factors. The ACm had a rather noisy signal, with a slightly increasing trend.

The variations in HR at different time scales, daily or over longer periods, seemed to be associated with different processes. Over some periods, for example, clear daily patterns in AC and HR appeared, with sudden increases in activity followed by a sharp increase in HR that progressively decreased until another peak of activity occurred (Figure 5). When the cage was fasted for more than 2 days, there was an increased variability of AC and TP (e.g., in late October; Figure 5).

**FIGURE 6:**
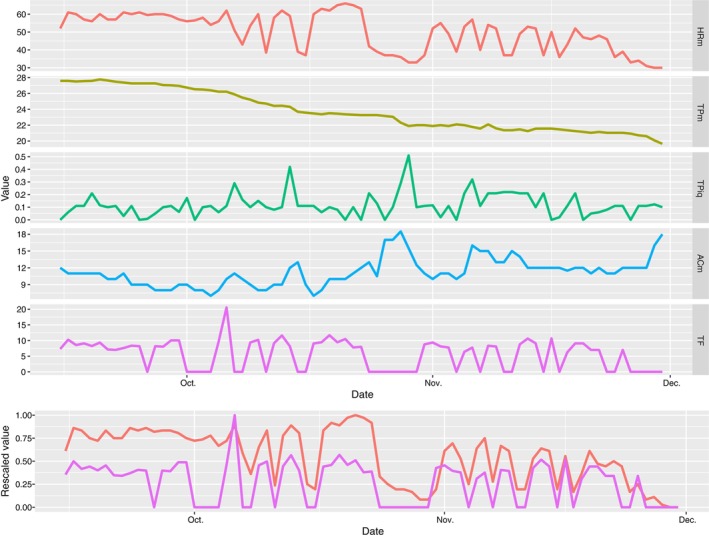
Time series of daily median values of heart rate (HRm, top panel), daily median values of peripheral body temperature (TPm, second panel), daily interquartile range of temperature (TPiq, third panel), daily median values of activity (ACm, fourth panel), and total food (TF, fifth panel). The bottom panel displays rescaled data of HRm and TF overlaid on top of each other.

Daily median values for each variable displayed clear patterns (Figure 6). HRm was stable until the beginning of October, when it started to become more variable, with a decreasing trend. It decreased from about 60 bpm to about 35 bpm over the study period, while TPm decreased from 27.5 to 19°C and there was increasing daily variability. The ACm seemed to increase slightly over the period whereas the TF displayed fluctuations that were very similar to those of HR, but without displaying a similar decreasing long‐term trend.

The nested modeling approach indicated that TF and TPm were explanatory variables for HRm. The best model DIC included a threshold effect of TPm on TF and a linear effect of TPm on HRm (Table 1). All models with this threshold effect of TPm on TF had significantly lower DIC and higher *R* ^2^ than the others. The TPm threshold in the best model was estimated to be 25.4°C (24.1–26.2) and the effect of food on HRm was not significantly different from 0 when the temperature was above that threshold, but was significantly positive below the threshold (Table 2). The effect of TPm on HRm was significantly positive as well.

**TABLE 1:** Model structure, effective number of parameters (pD), and deviance information criterion deviation (ΔDIC) compared to best model and *R* ^2^ for each of the model tested

Model	pD	ΔDIC	*R* ^2^
α + TF:thresh + TPm	5.6	0	0.815
α + TF:thresh + TPm + TPiq	5.9	1	0.812
α + TF:thresh + TPm + TPiq + ACm	7.2	3	0.808
α + TPiq:thresh + TF + TPm + ACm	4.4	13	0.725
α + ACm:thresh + TF + TPm	4.5	13	0.725
α + TF + TPm	5.5	14	0.723
α + TF + TPm + TPiq	3.6	14	0.711
α + TF + TPm + TPiq + ACm	3.4	14	0.708
α + TPiq:thresh + TF + TPm	5.2	14	0.722
α + TPm:thresh + TF	5.9	14	0.731
α + TPm:thresh + TF + TPiq + ACm	5.7	15	0.721
α + TPm:thresh + TF + TPiq	3.8	15	0.708
α + ACm:thresh + TF + TPm + TPiq	5.5	15	0.719
α + ACm:thresh + TF	6.1	25	0.664
α + TF:thresh	4.2	36	0.587
α + TF	2.1	52	0.51
α + TPiq:thresh + TF	3.6	53	0.512
α + TPm:thresh	8.5	72	0.429
α + ACm:thresh	4.9	87	0.323
α	1.3	141	−0.007
α + TPiq:thresh	5.2	141	0.017

**TABLE 2:** Parameters estimated for the best model

Parameter	Estimate	Standard Deviation	2.5%	97.5%
α	50.188	0.838	48.507	51.772
β^+^_TF_	0.313	0.338	−0.364	0.967
β^−^_TF_	1.831	0.211	1.432	2.237
β_TP_	2.137	0.346	1.462	2.810
Threshold	25.462	0.546	24.080	26.206

**FIGURE 7:**
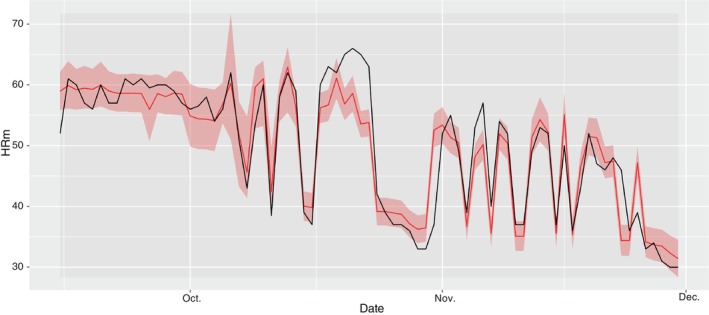
Predicted HRm values obtained from the best model (red) and observed HRm values (black). The pink area represents the 95% confidence interval.

The best model explained 81% of variance and its predictions captured most of the substantial variations in HRm while still being quite parsimonious (Figure 7).

## 4 DISCUSSION

The rapid subopercular deployment technique for HR‐tags, using a trocar, seems very promising in terms of consistent logging of good quality ECGs and HR data, and animal survival. The quality of data collection cannot be monitored during logging so it is essential to have a technique that yields meaningful information. The technique allows the HR‐tag to be placed very close to the heart, consistent with the literature on optimal locations for collecting good ECG signals (Brijs et al., 2018). The entry point for the trocar is easy to identify in relation to the operculum, which allows deployments to be repeated accurately and consistently. Furthermore, because the tag is advanced deep into the tissue it is presumably less likely to move; in Experiment 3 the tag remained in place for 80 days, despite an absence of stitches at the point of entry. Inspection showed that this entry point healed very well. As a consequence, good‐quality data were obtained over the entire experiment. The whole tagging procedure only required a few seconds and, since the deployment was intramuscular, the placement was relatively easy to control. This should ensure that the technique is reproducible and causes limited bleeding, being significantly less invasive than internal deployments in the body cavity, which require incisions and sutures, may cause injury to other organs, and have a risk of postsurgery complications such as infections. Therefore, this tagging technique is also less likely to affect fish health and behavior. Finally, the rapid subopercular technique allows easy deployment of HR‐tags on very large individuals (e.g., the 280 kg tuna in Experiment 3) for which more complex surgical interventions are logistically challenging.

The first and second experiments were conducted on the same day to show that the technique could be applied on living ABFT, that it provided useful ECG signals, and that ABFT survived following implantation. The ECGs that were recorded during Experiment 1 were, however, more noisy than in Experiment 3. This may have been partially because these were the first ever attempts at using this technique, but also because they were acute measurements on ABFT that were either brain dead (HR‐tag1) or moribund (HR‐tag2 and HR‐tag3), and air‐exposed on the deck of the ship. Experiment 2 had a very small sample size, constrained by the number of survival tags we possessed, which does not permit very firm conclusions about the effect of the technique on survival and behavior. Nonetheless, the tag implantation did not cause premature death and the distances subsequently traveled by implanted and nonimplanted fish were similar. Experiment 3 confirmed that implanted ABFT did not show premature mortality.

The HR and ECG data in Experiment 3 were much less noisy than those collected in Experiment 1 (Figure 3). This could be because the tag in Experiment 3 was custom‐made for ABFT, with a longer distance between the electrodes, which may improve the overall quality of the signal by increasing the signal‐to‐noise ratio. The fish was also alive and able to recover from handling stress and heal any tissue wounding during implantation. Our analysis of the data from Experiment 3 was not aimed at answering a specific ecological or physiological question. The objective was to illustrate the type of data that can be collected, its quality, and that it contained signals that could be interpreted in light of environmental conditions, notably temperature and feeding.

The variations in HR, ranging between 8 and 120 bpm, with 90% of the data between 28 and 70 bpm, are consistent with heart data obtained on smaller individuals (all below 21 kg) of other bluefin tuna species (Blank et al., 2004; Clark et al., 2008, 2013). The data clearly showed an overall positive association of HR with temperature but work on more individuals is needed to understand the validity and the basis of a threshold around 25°C, above which daily variations in HR linked to feeding were not visible. As the temperature decreased progressively over the 80 days, it was at experimental outset that the fish showed no daily fluctuations in HR. The fact that these became visible about 3 weeks later could also indicate recovery of normal cardiac activity after implantation. However, a 25 day recovery time seems unlikely because, in other teleost species, HR recovers from highly invasive surgical tag implantation in days rather than weeks (Brijs et al., 2019; Campbell, 2004; Gamperl et al., 2021; Mignucci et al., 2021). In Pacific bluefin tuna *Thunnus orientalis*, animals resume feeding within a matter of days after surgical implantation of temperature loggers in the visceral cavity (Gleiss et al., 2019), which is an indication of effective recovery.

The threshold around 25°C may, instead, be linked to an aspect of ABFT thermal physiology. This temperature is at the warm end of the waters that adult ABFT are reported to occupy in the wild, although they will inhabit waters up to 30°C during the breeding season (Block et al., 2001; Fromentin & Powers, 2005). Wild animals can, however, use dives below the thermocline to cool off (Block et al., 2001), something that caged fish cannot do. The threshold may have occurred in the caged fish because, above this temperature, it had high oxygen demand that required constant swimming activity to ram ventilate the gills. This may have obscured any variation in daily mHR associated with, notably, total food. Feeding is expected to raise HR because of the specific dynamic action (SDA) response, the metabolic cost of feeding, digestion, and assimilation that is a major component of the energy budget of fishes (Dupont‐Prinet et al., 2009; Fitzgibbon et al., 2007; McCue, 2006), with HR responses to feeding potentially more pronounced in bluefin tuna if they also swim faster to increase ram ventilation of the gills (Clark et al., 2008; Fitzgibbon et al., 2007; Gleiss et al., 2019). At the same time, the absence of variation in daily mHR rate above 25.5°C could also indicate that the animals were not feeding at all because they were physiologically constrained in their ability to meet the combined oxygen demands of warmth and specific dynamic activity (Whitlock et al., 2015).

The close association of daily mHR with total food, below the threshold value of 25.5°C, presumably reflects SDA responses. The SDA can itself raise HR due to increased metabolic oxygen demand (Dupont‐Prinet et al., 2009; Iversen et al., 2010) but the close association with total food may also reflect increased swimming activity to increase rates of gill ventilation (Clark et al., 2008; Fitzgibbon et al., 2007; Gleiss et al., 2019). This is because the swimming has a cost itself, which will also require increased HR and thereby enhance an association of HR to feeding in bluefin tuna species. Interestingly, although the ACm displayed some daily peaks that corresponded to increases in HRm, there was no overall and clear association between these two variables and average activity was not retained in the best model. In our data, the link between total food and average activity was somewhat at odds; in late October a period with no feeding at all was associated with an increase in average activity. A period of no feeding together with variability in temperature may explain increased average activity if the animals were searching for food rather than just schooling at some routine speed. Although all of this is speculation at this stage, the data do indicate the kind of insights that monitoring HR can provide in terms of revealing how environmental factors can influence physiology and behavior, including the identification of thermal thresholds.

In conclusion, the proposed technique allows HR‐tags to be implanted rapidly in large ABFT using a technique that is significantly simpler than the more invasive and time‐consuming surgical deployments in the body cavity (Clark et al., 2008). This technique does not allow data on internal temperature to be obtained but it has the advantages that it is rapid, minimally invasive, and provides high‐quality ECG data over extended periods of time. The technique might be specific to ABFT because it takes advantage of the species' skeletal anatomy and its size, which allow it to withstand implantation of relatively large tags by trocar. Therefore, this specific subopercular technique would need to be tested on other tuna species. Nonetheless, rapid implantation by trocar of archival tags may be a useful approach for many large pelagics under field conditions, with the actual procedure of tag placement requiring validation for a given species.

## AUTHOR CONTRIBUTIONS

T. Rouyer: designed the research and the technique, did the fieldwork, made the analyses and wrote the paper. S. Bonhommeau: designed the research and wrote the paper. S. Bernard, V. Kerzerho and O. Derridj: designed the technique and did the fieldwork. A. Bjarnason: designed the tags and made the analyses. H. Allal and J.F. Steffensen: designed the technique and wrote the paper. S. Deguara did the fieldwork and wrote the paper. B. Wendling: did the fieldwork and wrote the paper. G. Bal did the analyses and wrote the paper. D. Thambithurai: did the fieldwork and did the analysis. D.J. Mckenzie: designed the research and the technique and wrote the paper.

## FUNDING INFORMATION

This study is part of the PROMPT project funded by France Filière Pêche.

## Supporting information


**Data S1.** Analyses for the ECG algorithm validation.
