# Supplementary material for: A novel protocol for rapid deployment of heart rate data storage tags in Atlantic bluefin tuna Thunnus thynnus reveals cardiac responses to temperature and feeding
Source: J Fish Biol. 2023 Aug 3;106(5):1305–15. doi: 10.1111/jfb.15507 (PMC12120331; doi:10.1111/jfb.15507)
Supplement: Supplementary file 1 — Data S1. Analyses for the ECG algorithm validation. [file JFB-106-1305-s001.docx]

**Electronic Supplementary Material (ESM)**

Content

Appendix 1: Additional methods

1.a Validation and filtering of heart rates

We visually inspected and validated 3789 ECG traces using HRT Analyzer software from Star-Oddi recorded with a custom made Centi-HRT ACT G2 70mm long data storage tag. With that it was possible to confirm if the HR was being calculated correctly or if manual annotation was needed. Each ECG trace is 7.5 second long sampled at 200Hz, the trace goes through on-board processing for heart rate and a quality estimation (QI). The distribution of manually validated values and those validated by the algorithm are shown on Figure S1.


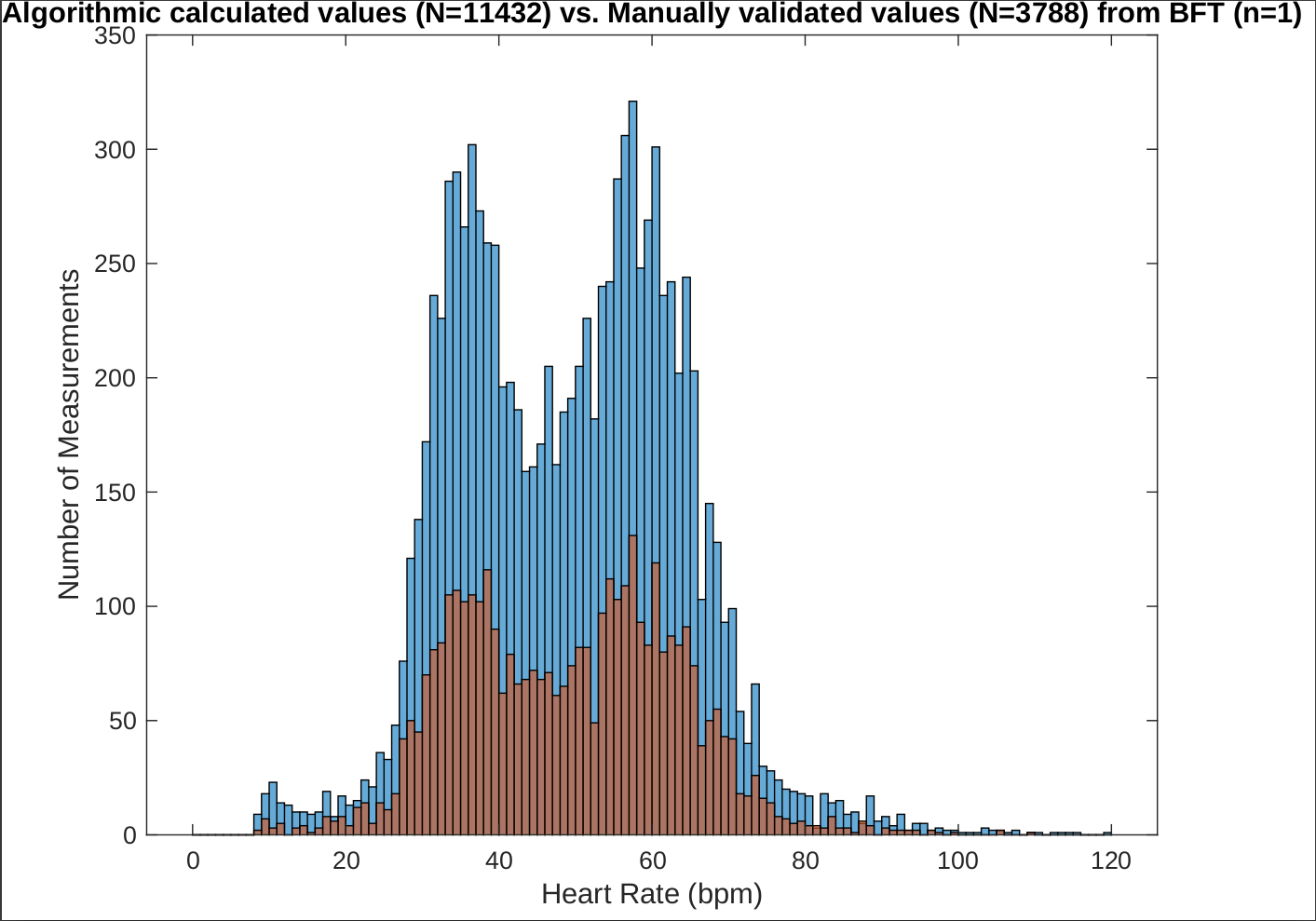


*Figure S 1: Distribution of manually validated Heart rate values (red) and algorithmic calculated (blue).*

Furthermore it is possible to validate highest and lowest ECG recorded heart rate, shown in Figure S2 namely 105bpm and Figure S3 8bpm which is also the lowest theoretical heart rate the logger can record in the setup used (7.5 seconds).


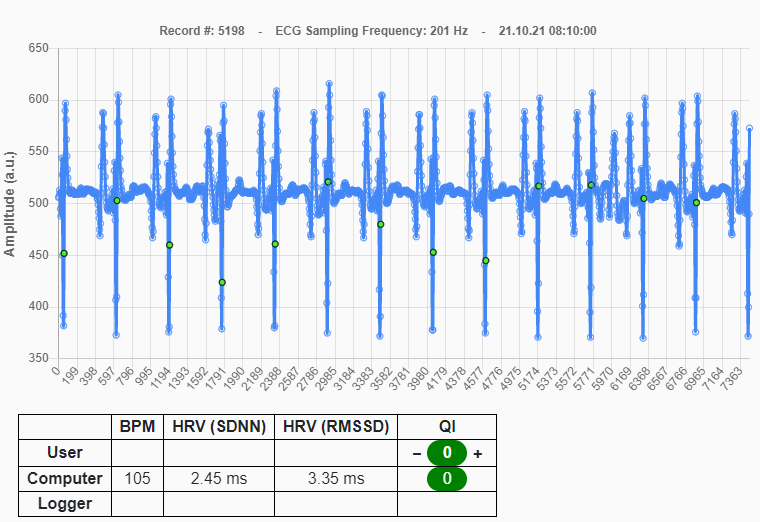


*Figure S 2: Highest heart rate recorded with ECG, 105 bpm*


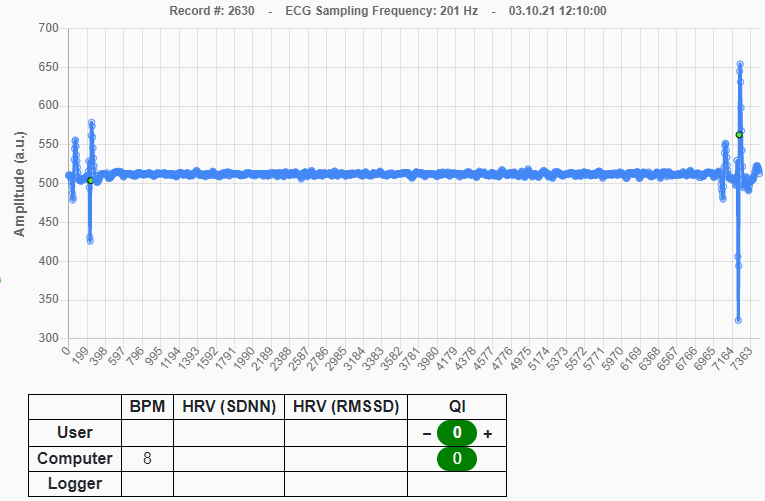


*Figure S 3: Lowest heart rate recorded with ECG, 8bpm. Just a single R-R interval.*

Figure S4 shows correlation between the recorded heart rate provided by the logger and the validated value for each of the four quality indices similar approach to (Trondrud et al 2021). The blue circles indicate records calculated by the logger from 8bpm to 120bpm which was selected as upper limit of heart rate for this ABFT.

Based on the R-squared values it was decided to include recordings with QI equal to 0 and 1 but to exclude measurements with QI 2 and 3.


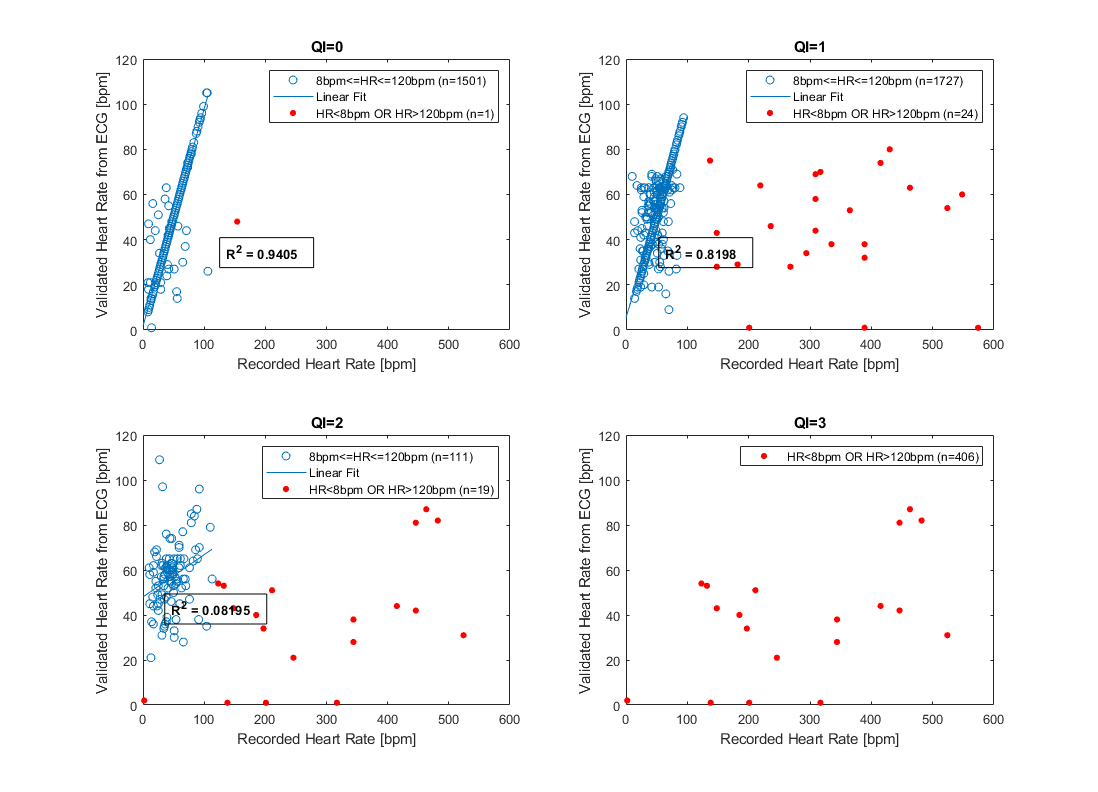


*Figure S 4: Correlation between validated HR calculations based on ECG and the recorded heart rate calculations of the logger. R-squared values are presented for heart rate in the range of 8 to 120bpm.*


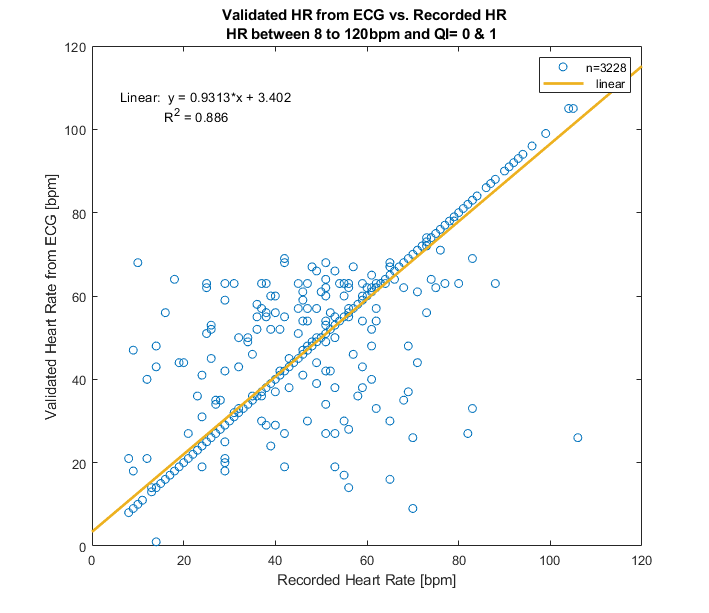


*Figure S 5: Correlation between validated HR and recorded heart rate by the logger. Representative of the complete HR dataset used in experiment 3.*

Citations

Trondrud, L. Monica; Pigeon, Gabriel; Albon, Steve; Arnold, Walter; Evans, Alina L.; Irvine, R. Justin; et al. (2021): Supplementary material from "Determinants of heart rate in Svalbard reindeer reveal mechanisms of seasonal energy management". The Royal Society. Collection. https://doi.org/10.6084/m9.figshare.c.5420046.v2
